# Phenotypic plasticity can facilitate adaptive evolution in gene regulatory circuits

**DOI:** 10.1186/1471-2148-11-5

**Published:** 2011-01-06

**Authors:** Carlos Espinosa-Soto, Olivier C Martin, Andreas Wagner

**Affiliations:** 1 University of Zurich, Dept. of Biochemistry, Bldg. Y27 Winterthurerstrasse 190 CH-8057 Zurich, Switzerland; 2 The Swiss Institute of Bioinformatics. Quartier Sorge, Batiment Genopode, 1015 Lausanne, Switzerland; 3 INRA, UMR 0320/UMR 8120 Génétique Végétale, F-91190 Gif-sur- Yvette, France; 4 The Santa Fe Institute, 1399 Hyde Park Road, Santa Fe, NM 87501, USA

## Abstract

**Background:**

Many important evolutionary adaptations originate in the modification of gene regulatory circuits to produce new gene activity phenotypes. How do evolving populations sift through an astronomical number of circuits to find circuits with new adaptive phenotypes? The answer may often involve phenotypic plasticity. Phenotypic plasticity allows a genotype to produce different - alternative - phenotypes after non-genetic perturbations that include gene expression noise, environmental change, or epigenetic modification.

**Results:**

We here analyze a well-studied model of gene regulatory circuits. A circuit's genotype encodes the regulatory interactions among circuit genes, and its phenotype corresponds to a stable gene activity pattern the circuit forms. For this model, we study how genotypes are arranged in genotype space, where the distance between two genotypes reflects the number of regulatory mutations that set those genotypes apart. Specifically, we address whether this arrangement favors adaptive evolution mediated by plasticity. We find that plasticity facilitates the origin of genotypes that produce a new phenotype in response to non-genetic perturbations. We also find that selection can then stabilize the new phenotype genetically, allowing it to become a circuit's dominant gene expression phenotype. These are generic properties of the circuits we study here.

**Conclusions:**

Taken together, our observations suggest that phenotypic plasticity frequently facilitates the evolution of novel beneficial gene activity patterns in gene regulatory circuits.

## Background

Novel adaptive phenotypes endow organisms with new means to survive and reproduce. Such new phenotypes arise through a process that involves natural selection and random genotypic change caused by mutation. Life's ability to adapt through random change is remarkable, as many man-made systems do not have this ability [1,2]. It is a result of how genotypic change translates into phenotypic change [1,3]. 

Different classes of biological systems, ranging from protein and RNA molecules [4-6] to regulatory circuits [7] and genome-scale metabolic networks [8], share some similarities in how they translate genotypic change into phenotypic change. First, any genotype *G *produces some phenotype *P *in the absence of environmental and other perturbations. We will refer to such a 'default' phenotype as *G*'s native phenotype (analogous to the native conformation of a protein). Second, in all these systems genotypes exist in vast genotype spaces. In a genotype space, the distance between two genotypes indicates the number of mutations that set those genotypes apart. Third, the set of genotypes with the same native phenotype define a "genotype network". For any two genotypes on a genotype network, there exists a sequence of small genetic changes that leads from one genotype to the other, without ever changing the native phenotype. Genotype networks are typically vast and extend far through genotype space. A population that evolves on a genotype network through mutation and selection can traverse large regions of genotype space, while the population's bulk preserves the same phenotype. While doing so, members of the population can explore different phenotypes that occur in different regions of genotype space. Because genotype networks extend far through this space, they facilitate exploration of many novel phenotypes [5,8-12]. In sum, genotype networks allow access to a wide range of new, potentially adaptive phenotypes [13,14].

Despite the existence of genotype networks, finding a specific novel phenotype through a blind evolutionary search is challenging, partly because genotype spaces are very large, partly because the fraction of advantageous novel phenotypes encountered during this search is usually small. For example, many more mutations are deleterious rather than advantageous [15,16]. Here we explore a phenomenon that can facilitate this search, that of phenotypic plasticity.

Phenotypic plasticity allows a genotype to produce more than one distinct phenotype [17-19]. Such alternative phenotypes are induced by non-genetic perturbations. Non-genetic perturbations influence the formation of all kinds of phenotypes, but molecular phenotypes illustrate this influence especially well. Consider protein structure phenotypes. Driven by thermal noise - a ubiquitous kind of non-genetic perturbation - the same amino acid sequence (genotype) typically folds into one main, native structure (phenotype), as well as a large spectrum of alternative structures. Several aspects of the protein's environment can influence which of these structures it forms. They include temperature, pH, but also other proteins, such as chaperones [20] or infective proteinaceous agents (prions) [21]. The same holds for RNA molecules, where a single genotype (nucleotide sequence) can also fold into different structures [6,22,23]. On higher levels of organization, genes and their products interact in regulatory circuits. The genotype determines which of a circuit's genes interact; the gene activity or gene expression phenotypes resulting from these interactions are, again, influenced by non-genetic factors. For instance, a circuit's native gene expression phenotype can be altered through stochastic change - intracellular noise - in the number of regulatory molecules inside a cell [24-26]. Biotic or abiotic environmental factors can also change a gene regulatory circuit's activity pattern and the macroscopic traits it helps build [17,19,27-29].

The genotype network concept can readily accommodate the phenomenon of phenotypic plasticity [22]. Genotypes that produce a given phenotype as their native phenotype belong to the same genotype network, but each of these genotypes may also produce a spectrum of alternative phenotypes. This spectrum may differ among genotypes on the same genotype network, and not all of these genotypes may have equal plasticity. For example, one genotype may readily produce an alternative phenotype, whereas in another genotype, the same phenotype may arise only rarely, for example through an extreme and rare perturbation. In these two genotypes, the phenotype would then have high and low *penetrance*, respectively. 

A growing body of work suggests that phenotypic plasticity strongly influences the origin of novel phenotypes [17-19,30-44]. The earliest support comes from classic work by Waddington [45,46], Schmalhausen [47] and Baldwin [48]. Waddington showed that artificial selection of a phenotype that initially appears only in a few organisms after non-genetic perturbations, can easily result in the trait's genetic determination [46,49]. More recently, other researchers have made the same observation for diverse traits and different species [37,38,42]. Artificial selection can thus turn an alternative into a native phenotype. In addition, many observations in wild populations suggest that in multiple cases an ancestral alternative phenotype may have facilitated the evolution of new, genetically fixed adaptive traits [17-19,30-36,40]. The phenotypes where plasticity may have facilitated adaptation are very diverse. They include gill surface area in cichlid fishes [33], pigmentation patterns in the crustacean *Daphnia melanica *[34], and head size in the snake *Notechis scutatus *[35], to name but a few. Despite an abundance of candidate examples, plasticity's importance for adaptive evolution is not universally accepted [50,51]. We still do not know whether existing observations from artificial selection experiments or from wild populations are rare oddities or hint at general principles of evolution [39,43,44,52,53].

**Figure 1:**
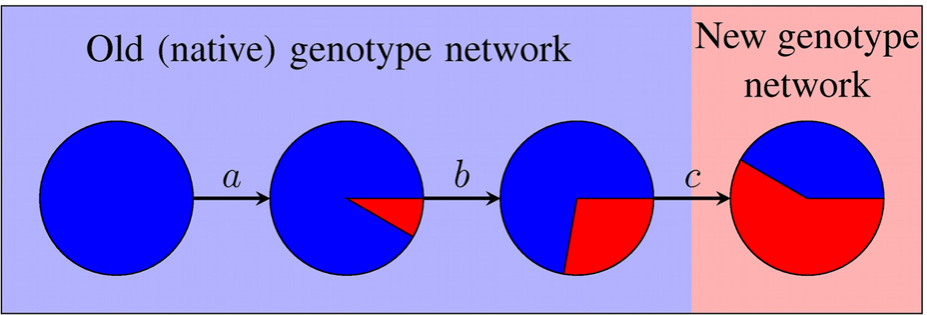
**A plasticity-mediated evolutionary path towards a new adaptive phenotype**. Each circle represents a genotype. Lines represent mutations that convert one genotype into another. Different colors represent distinct phenotypes. The same background color surrounds genotypes in the same genotype network. Colored areas within circles represent the probability that a genotype produces a particular phenotype. Blue represents an original native phenotype, and red a new beneficial phenotype. We show a sequence of mutations towards the red genotype network. First, the red phenotype arises as a low penetrance alternative phenotype (mutation *a*). As penetrance increases, the distance to the new genotype network decreases (*b*). Finally, the new phenotype has become stabilized, that is, a new genotype network has been 'discovered' (*c*).

If important for adaptive evolution, plasticity would facilitate adaptation through a scenario such as the following (Figure 1): Consider a population in 'search' of some new superior phenotype *P_new_*. At some point, genotypes arise that have *P_new _*as a low-penetrance member of their spectrum of alternative phenotypes. Such genotypes would accumulate through selection, as they occasionally produce *P_new_*. Second, some mutations in these genotypes produce genotypes where *P_new _*has higher penetrance. These mutant genotypes now accumulate in the population. Finally, the population gains mutational access to *P_new_*'s genotype network. In this genotype network *P_new _*is produced as the native phenotype - without any non-genetic perturbations. Here, *P_new _*has become genetically stabilized.

In the above scenario, genotypes that produce a phenotype *P_new _*through plasticity have better chances to find *P_new_*'s genotype network. This scenario requires several properties of the organization of genotype space. The conditions are:

i) Finding genotypes that can produce a new alternative phenotype *P_new _*through plasticity must be significantly easier than finding the genotype network of *P_new _*(i.e. genotypes where *P_new _*is native).

ii) Genotypes near the genotype network of *P_new _*should have a tendency to produce *P_new _*as an alternative phenotype. Otherwise, reaching *P_new_*'s genotype network would not be easier from genotypes that produce *P_new _*through plasticity.

iii) Mutations of genotypes that produce *P_new _*through plasticity must often result in genotypes that can also produce *P_new _*through plasticity. Otherwise, an evolutionary search of *P_new_*'s genotype network might not be able to progress through genotypes that can all produce *P_new_*.

iv) The higher the penetrance of *P_new _*is in a given genotype *G*, the easier it must be to reach *P_new_*'s genotype network from *G*.

Conditions i)-iii) imply that there must be sets of mutationally connected genotypes that produce *P_new _*as an alternative phenotype. From some of those genotypes, a single mutation must suffice to reach *P_new_*'s genotype network. Condition iv) implies that selection can promote genetic stabilization of *P_new _*through gradual increases in *P_new_*'s penetrance.

We here explore whether these conditions are fulfilled in a model of transcriptional regulation circuits. Such circuits produce specific gene activity patterns in different parts and developmental stages of an organism. In doing so, they have a major role in directing developmental processes [54,55]. Many evolutionary novelties involve changes in the gene expression patterns such circuits produce [56-59]. In the circuits we study, the genotype encodes the transcriptional regulatory interactions determined by a circuit's cis-regulatory regions. A phenotype corresponds to a circuit's stable gene activity pattern. We show that the requirements we mentioned above are fulfilled for these circuits. Our work suggests that phenotypic plasticity can facilitate adaptive evolution that involves changes in gene activity patterns of regulatory circuitry.

## Results

### The model

Our model represents gene regulatory circuits comprising *N *genes. The activity of each gene in a circuit is regulated by the activity of other circuit genes. An *N *by *N *real-valued matrix **A **= (*a_ij_*) specifies the manner in which this regulation occurs. We view this matrix as a circuit's regulatory genotype. A gene *j *regulates the activity of another gene *i *when *a_ij _*≠ 0. The effect of gene *j *on gene *i *can be either activating (*a_ij _*> 0) or repressing (*a_ij _*< 0). We call two circuits neighbors (in a regulatory genotype space) if they differ in a single regulatory interaction. We use the integer variable *m *to denote a circuit's number of regulatory interactions, i.e. the number of non-zero values in **A**; we use the real number *c *to denote a circuit's interaction density, that is, its number of interactions *m *divided by the maximally possible number of interactions *N*^2^. We describe the activity state of the circuit at time *t *with a vector $s_{t}=(s_{t}^{\left(1\right)},...,s_{t}^{\left(N\right)})$. In our model, cross-regulatory interactions among circuit genes and the circuit's activity state at time *t *determine the circuit's activity state after a time-step of length τ as follows:

(1) $$s_{t+\tau }^{\left(i\right)}=\sigma \left[\sum_{j=1}^{N}a_{ij}s_{t}^{\left(j\right)}\right]$$

where σ(*x*) equals -1 when *x *< 0, it equals +1 when *x *> 0, and it equals 0 when *x *= 0.

Despite this model's simplicity, it has been successfully used to study various aspects of the evolution of gene regulatory circuits, such as the evolution of robustness, of modularity, and of pattern formation [7,11,60-66]. Variants of the model have also proven useful to model developmental processes in plants and animals [67,68].

We here consider circuits that attain a stable gene activity pattern *s_∞ _*when their dynamics start from an initial gene activity pattern *s*_0_. Such an initial state is determined by factors outside the circuit, be they genes 'upstream' of the circuit, maternal regulators, signals from neighboring cells or environmental factors. We refer to a stable gene activity pattern *s_∞ _*as a gene activity *phenotype*. As in previous research [7], we do not analyze circuits that fail to produce a stable activity pattern.

Circuit genotypes with the same gene activity phenotype form vast genotype networks in a space of regulatory circuits [7]. Throughout this paper, we consider circuits in a given genotype network, that is, they attain a given gene activity phenotype $s_{\infty }^{native}$ from a given initial gene activity state s_0 _through the circuit's dynamics. We refer to this genotype network as the 'native genotype network' and to $s_{\infty }^{native}$ as the 'native phenotype'. The activity state s_0 _is the gene activity state from which the system starts its dynamics in the absence of non-genetic perturbations. We note that all properties of genotype network organization relevant to us depend on the fraction *d *of gene activity differences between *s*_0 _and $s_{\infty }^{native}$, and not on the identity of these activity patterns [7].

We study two kinds of perturbations. The first is a mutation of a circuit's regulatory genotype. A mutation changes an interaction by altering a value of *a_ij _*in a circuit's matrix **A**. Some mutations can cause a circuit to produce a phenotype different from $s_{\infty }^{native}$. The second kind of perturbation has a non-genetic origin and affects the initial gene activity pattern s_0_. Such a perturbation could result, for example, from intracellular noise, from environmental fluctuations, or from disturbances in the activity of genes upstream of the circuit. For example, intracellular noise can create gene expression heterogeneity in clonal populations [24-26], just as exposure to some environmental factors can induce major gene expression changes in different organismal lineages [27-29], and impairing the activity of pair-rule genes upstream of the segment-polarity gene circuit in fruit flies can change the expression pattern of genes in this circuit [69,70]. Such perturbations can alter developmental trajectories, and result in new gene activity phenotypes different from $s_{\infty }^{native}$.

We call the phenotypes that a circuit genotype *G *produces after non-genetic perturbations and that are different from *G*'s native phenotype 'alternative phenotypes'. Some alternative phenotypes may be detrimental, but others may be beneficial [40,71,72]. An alternative phenotype has a low (high) penetrance if the likelihood that *G *produces it after a random perturbation in the initial condition is low (high).

### Finding new alternative phenotypes is easier than finding new native phenotypes

**Figure 2:**
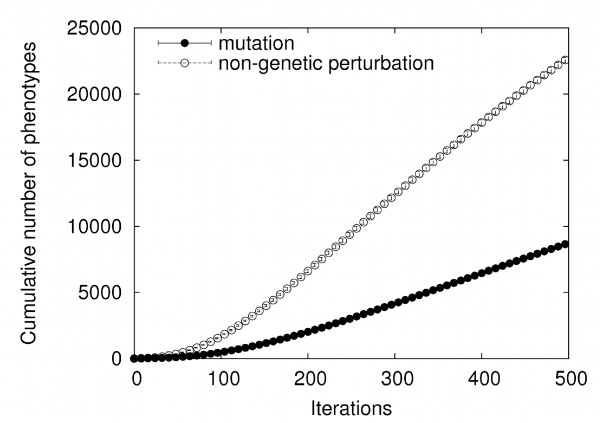
**Gene circuits exploring a genotype network find genotypes with novel alternative phenotypes faster than new genotype networks**. The figure shows mean values for 200 independent ensembles of circuits. Ensemble size *M *= 5 × 10^3 ^circuits with *N *= 16 genes, interaction density *c *≈ 0.35, and a fraction *d *of gene activity differences between *s*_0 _and $s_{\infty }^{native}$ equal to 0.125.

We first asked whether mutation-driven exploration of a genotype network can find new alternative phenotypes more easily than new genotype networks (native phenotypes), as required by condition i) in the introduction. To answer this question, we allowed an ensemble of 5 × 10^3 ^circuits to drift randomly on a genotype network by changing one regulatory interaction at a time, while preserving the circuits' native phenotype. During this process, we recorded two observables. The first was the *cumulative *number of new phenotypes that circuits could produce after each possible single gene-activity modifications of *s*_0 _(i.e. non-genetic perturbations). That is, after each mutation, we determined those alternative phenotypes that a mutated genotype could produce but that previous genotypes had not been able to produce, and appended these alternative phenotypes to a growing list of such phenotypes. The second observable was the cumulative number of new phenotypes that the circuits explored exclusively through mutation. Every time a mutation caused a change in a circuit's native phenotype, we recorded the new phenotype, before replacing the circuit by its parent in the original native genotype network. We appended these new phenotypes to a growing list of phenotypes that had not been encountered through previous mutations. In this analysis, we found that plasticity allows a faster exploration of new phenotypes than mutation alone. Figure 2 shows that after each circuit in the ensemble had experienced 500 mutations, plasticity had explored more than twice as many phenotypes as mutation. This figure averages results for 200 independent ensembles of circuits with *N *= 16 genes, interaction density *c *≈ 0.35, and a fraction *d *of gene activity differences between *s*_0 _and $s_{\infty }^{native}$ equal to 0.125. Circuits with different values of these parameters also have faster access to new phenotypes through plasticity than through mutation alone (Additional file 1: Figure S1).

### Mutations and non-genetic perturbations produce similar sets of phenotypes

If phenotypic plasticity facilitates the discovery of new genotype networks, then mutations and non-genetic change should often produce similar or identical new phenotypes (condition ii) in the introduction). Otherwise, genotypes that produce an alternative phenotype after non-genetic perturbations would not have preferential mutational access to that phenotype's genotype network. In other words, if condition ii) did not hold, mutations would not be able to turn an alternative phenotype into a native phenotype that is produced even in the absence of non-genetic perturbations. To find out whether this is the case, we sampled random genotypes from a given genotype network. All the genotypes on this network produce the same phenotype $s_{\infty }^{native}$ from *s*_0_. We defined the following sets for each genotype *G *in the sample: *P_μ _*is the set of new phenotypes that mutations in *G *can create; $P_{s_{0}}$ is the set of alternative phenotypes that perturbation of each gene in the initial condition *s*_0 _can create. To quantify the similarity between both sets, we defined the index *C *as the size of the intersection between both sets divided by its maximally possible value for sets of the same size $\left(C=\frac{\left|P_{\mu }\cap P_{s_{0}}\right|}{\min(|P_{\mu }|,|P_{s_{0}}|)}\right)$. *C *ranges from zero, if mutation and non-genetic perturbations produce always different phenotypes, to one, if *P_μ _*and $P_{s_{0}}$ fully overlap. We also defined an index *C^rand^*, which estimates how similar randomly chosen sets of phenotypes would be (see Methods). By assessing *C*, we found that mutations and non-genetic perturbations produce the same phenotypes more often than expected by chance (Additional file 2: Figure S2). This observation holds for all combinations of circuit sizes *N*, interaction densities *c*, and distances *d *between *s*_0 _and $s_{\infty }^{native}$ we examined (Table 1; Wilcoxon signed-rank test; *p *< 2.2 × 10^-16 ^in all cases). Thus, genotypes that require a single mutation to reach the genotype network of a phenotype *P_new _*also tend to produce *P_new _*as an alternative phenotype.

**Table 1:** Mutations and perturbations in the initial condition s0 produce the same phenotypes more often than expected by chance

*N*	*c*	*d*	**Mean *C *± S.E**.	*p*-value	Sample size*^a^*
8	0.4	0.125	0.528 ± 0.006	< 2.2 × 10^-16^	5485
		
		0.25	0.496 ± 0.006	< 2.2 × 10^-16^	5905
		
		0.5	0.557 ± 0.005	< 2.2 × 10^-16^	7269
	
	0.3	0.125	0.442 ± 0.006	< 2.2 × 10^-16^	5885
		
		0.25	0.398 ± 0.006	< 2.2 × 10^-16^	6050
		
		0.5	0.349 ± 0.005	< 2.2 × 10^-16^	7144

20	0.3	0.1	0.712 ± 0.006	< 2.2 × 10^-16^	5247
		
		0.25	0.8 ± 0.005	< 2.2 × 10^-16^	6432
		
		0.5	0.885 ± 0.003	< 2.2 × 10^-16^	8055
	
	0.2	0.1	0.63 ± 0.006	< 2.2 × 10^-16^	5318
		
		0.25	0.645 ± 0.006	< 2.2 × 10^-16^	5699
		
		0.5	0.711 ± 0.005	< 2.2 × 10^-16^	7102
	
	0.1	0.1	0.47 ± 0.006	< 2.2 × 10^-16^	6036
		
		0.25	0.408 ± 0.006	< 2.2 × 10^-16^	6058
		
		0.5	0.364 ± 0.005	< 2.2 × 10^-16^	6776

*C *>*C^rand^*, according to a Wilcoxon signed-rank test. In this and all other tables, *N *refers to the number of genes in a circuit, *c *refers to the circuit's interaction density, and *d *refers to the fraction of gene activity differences between the unperturbed initial condition *s*_0 _and the native phenotype $s_{\infty }^{native}$.

^*a *^Even though we sampled 10^4 ^genotypes for each genotype network, we discarded genotypes in which either *P_μ _*or $P_{s_{0}}$ was empty.

### Similar circuits produce a similar spectrum of alternative phenotypes

Genotypes producing a new phenotype $s_{\infty }^{new}$ through plasticity, but that are not themselves neighbors of $s_{\infty }^{new}$'s genotype network may still aid in the genetic stabilization of $s_{\infty }^{new}$. This could occur if neighboring genotypes typically produce the same alternative phenotypes [condition iii)]. In this case, mutations that preserve $s_{\infty }^{new}$ as an alternative phenotype can make it easier to reach $s_{\infty }^{new}$'s genotype network.

Motivated by these considerations, we asked whether similar circuits produce similar sets of alternative phenotypes. We use the symbol $P_{s_{0}}$ to refer to the set of alternative phenotypes that a circuit genotype *G *produces after non-genetic perturbations. $P_{s_{0},k}$ refers to the set of alternative phenotypes that a genotype differing from *G *in *k *regulatory interactions, but residing on the same genotype network produces. We define the index $C_{k}=\frac{\left|P_{s_{0}}\cap P_{s_{0},k}\right|}{\min(|P_{s_{0}}|,|P_{s_{0},k}|)}$. It varies from zero to one as the two sets of alternative phenotypes range from completely disjoint to fully overlapping.

**Figure 3:**
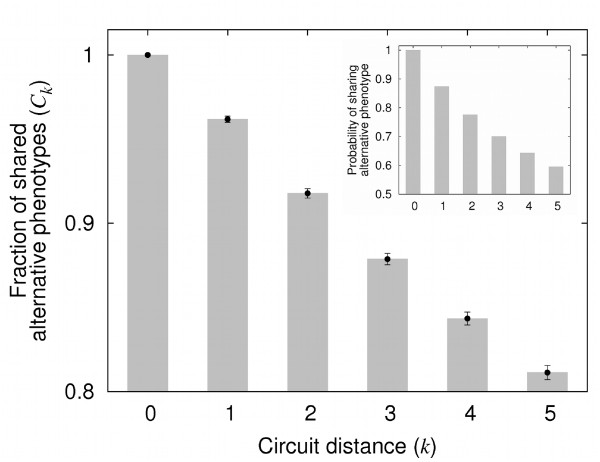
**The more similar two circuit genotypes are, the higher the overlap between their sets of alternative phenotypes**. The number *k *represents the distance (in the number of differing regulatory interactions) between two circuits. *C_k _*measures the similarity between the sets of alternative phenotypes produced by the two circuits. The plot shows mean values and the length of error bars represents one standard error. Spearman's *ρ *= -0.248; *p *< 2.2 × 10^-16^. The inset shows that the fraction of genotype pairs that share a given alternative phenotype decreases with *k*. Data are based on 10^4^ randomly sampled focal circuits with *N *= 20, *c *≈ 0.2, and *d*= 0.1.

Similar circuit genotypes produce similar sets of alternative phenotypes (Figure 3). Genotypes that differ in a single mutation share, on average, more than 93% of their alternative phenotypes (*C*_1 _> 0.93). High similarity in alternative phenotypes also holds after variation on values of *N*, *c *and *d *(Table 2). Figure 3 also illustrates how *C_k _*decreases as two circuits diverge. Circuit distance *k *and *C_k _*are negatively associated for all values of *N*, *c *and *d *that we examined (Table 2; Spearman's *ρ*; *p *< 2.2 10^-16 ^in all cases).

**Table 2:** The higher the similarity between two circuits is, the higher is the overlap between their sets of alternative phenotypes

*N*	*c*	*d*	Mean *C*_1 _± S.E. (Sample size*^a^*)	Spearman's *ρ*	*p*-value
8	0.4	0.125	0.95 ± 0.002 (9124)	-0.279	< 2.2 × 10^-16^
		
		0.25	0.956 ± 0.002 (9248)	-0.247	< 2.2 × 10^-16^
		
		0.5	0.968 ± 0.002 (9499)	-0.193	< 2.2 × 10^-16^
	
	0.3	0.125	0.933 ± 0.002 (9408)	-0.295	< 2.2 × 10^-16^
		
		0.25	0.94 ± 0.002 (9459)	-0.27	< 2.2 × 10^-16^
		
		0.5	0.963 ± 0.002 (9649)	-0.206	< 2.2 × 10^-16^

20	0.3	0.1	0.968 ± 0.002 (8712)	-0.237	< 2.2 × 10^-16^
		
		0.25	0.97 ± 0.002 (8910)	-0.201	< 2.2 × 10^-16^
		
		0.5	0.98 ± 0.001 (9403)	-0.153	< 2.2 × 10^-16^
	
	0.2	0.1	0.962 ± 0.002 (9216)	-0.248	< 2.2 × 10^-16^
		
		0.25	0.965 ± 0.002 (9179)	-0.214	< 2.2 × 10^-16^
		
		0.5	0.977 ± 0.001 (9440)	-0.178	< 2.2 × 10^-16^
	
	0.1	0.1	0.948 ± 0.002 (9675)	-0.255	< 2.2 × 10^-16^
		
		0.25	0.957 ± 0.002 (9696)	-0.237	< 2.2 × 10^-16^
		
		0.5	0.966 ± 0.002 (9748)	-0.189	< 2.2 × 10^-16^

This overlap decreases with the genetic distance between the circuits, according to Spearman's *ρ*, a non-parametric rank correlation coefficient [76]. *C*_1 _is the overlap in the sets of alternative phenotypes of two genotypes that differ in a single regulatory interaction.

*^a ^*We sampled 10^4 ^independent genotypes for each parameter combination, but we discarded genotypes that differ in *k *interactions where *P*_*s*0,*k *_was empty.

In addition, for each of 10^4 ^circuits on the same genotype network, we picked randomly one of the circuit's alternative phenotypes. We asked whether genotypes in the same genotype network but differing from the focal genotype in *k *regulatory interactions also produced the alternative phenotype through plasticity. More than half of the genotypes that differ from the focal genotype by five or fewer mutations also produced the same alternative phenotype (inset in Figure 3 and Table 3). For all values of *N*, *c *and *d *we examined, the probability that two genotypes produce the same alternative phenotype was above 0.8 for genotypes that differ in a single regulatory interaction (Table 3). This probability decreases with the number *k *of regulatory interactions in which two circuits differ (Figure 3 and Table 3). In sum, similar circuits have better odds to produce the same alternative phenotype. Thus, genotypes that produce an alternative phenotype $s_{\infty }^{new}$ but that are not neighbors of $s_{\infty }^{new}$'s genotype network can have indirect mutational access to this network. Other genotypes that can produce $s_{\infty }^{new}$ as an alternative phenotype may provide this access, enabling genetic stabilization of the new phenotype.

**Table 3:** The probability that two circuit genotypes share an alternative phenotype decreases with their genotypic distance

*N*	*c*	*d*	**Fraction of genotypes that share a specific phenotype after *k *mutations**.				
			
			*k *= 1	*k *= 2	*k *= 3	*k *= 4	*k *= 5
8	0.4	0.125	0.854	0.749	0.664	0.593	0.546
		
		0.25	0.874	0.777	0.706	0.645	0.6
		
		0.5	0.911	0.839	0.786	0.748	0.713
	
	0.3	0.125	0.869	0.768	0.691	0.624	0.568
		
		0.25	0.882	0.787	0.716	0.657	0.606
		
		0.5	0.925	0.863	0.809	0.765	0.727

20	0.3	0.1	0.826	0.718	0.644	0.59	0.546
		
		0.25	0.85	0.753	0.687	0.646	0.619
		
		0.5	0.907	0.849	0.804	0.774	0.75
	
	0.2	0.1	0.874	0.776	0.701	0.643	0.596
		
		0.25	0.877	0.782	0.719	0.663	0.617
		
		0.5	0.913	0.85	0.799	0.762	0.73
	
	0.1	0.1	0.913	0.848	0.784	0.728	0.676
		
		0.25	0.924	0.857	0.797	0.743	0.701
		
		0.5	0.939	0.891	0.849	0.811	0.779

### Genetic distance to a new genotype network is negatively correlated to a phenotype's penetrance

Thus far, we demonstrated that conditions i) through iii) of our evolutionary scenario hold. We now turn to condition iv). This condition requires that circuits with a high penetrance alternative phenotype *P_new _*have preferential (mutational) access to *P_new_*'s genotype network, where this phenotype is native. We next show in several complementary ways that this is the case.

**Figure 4:**
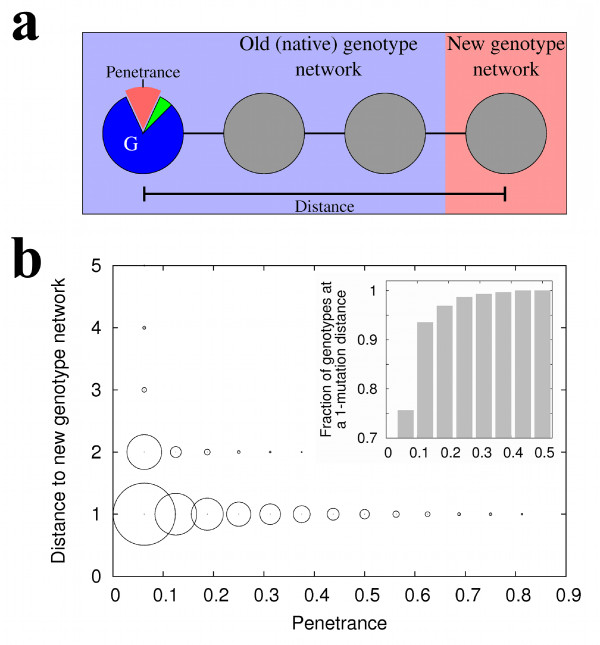
**Genetic distance to a genotype network is negatively correlated with a phenotype's penetrance**. (a) The leftmost circle represents a genotype *G*, where an alternative phenotype $s_{\infty }^{new}$ has a given penetrance (red sector). We quantified the penetrance of $s_{\infty }^{new}$ in *G *as the fraction of single-gene perturbations of the initial condition *s*0 that produce $s_{\infty }^{new}$. We determined genetic distance as the smallest number of mutations required to reach $s_{\infty }^{new}$'s genotype network (rightmost circle). (b) The horizontal axis shows the penetrance of a phenotype $s_{\infty }^{new}$. The vertical axis shows the distance to $s_{\infty }^{new}$'s genotype network. A circle's area is proportional to the number of data points in each penetrance and mutational distance category. The panel is based on 10^4 ^circuit genotypes with *N *= 16 genes, *c *≈ 0.35, and *d *= 0.25. Penetrance and genetic distance to the new genotype network are negatively associated (Spearman's *ρ *= -0.293; *p *< 2.2 × 10^-16^). The inset shows that the fraction of genotypes that are neighbors of the new genotype network increases with penetrance.

We quantified the penetrance of a given phenotype as the fraction of different single-gene expression perturbations in a circuit's initial state that produce this phenotype. We then assessed whether a phenotype's penetrance is linked to a circuit's proximity to a new genotype network, as follows (Figure 4a). We first chose a genotype *G *at random among all genotypes in a pre-determined genotype network. Second, we determined *G*'s alternative phenotypes and their penetrance. Third, we chose one of the alternative phenotypes at random, regardless of its penetrance, and called it $s_{\infty }^{new}$. Fourth, we estimated the distance of *G *to the genotype network of $s_{\infty }^{new}$ (see Methods). We repeated this procedure for 10^4 ^genotypes for each combination of values of *N*, *c *and *d *that we examined.

Figure 4b illustrates our findings. The horizontal axis shows a phenotype's penetrance, and the vertical axis the distance to the new genotype network. The area of each circle reflects the number of genotypes in each penetrance/mutational distance category. Starting from a genotype that produces an alternative phenotype $s_{\infty }^{new}$, one to two mutations are generally sufficient to reach $s_{\infty }^{new}$'s genotype network. Figure 4b also shows that distance to a new genotype network decreases with increasing penetrance. The inset focuses on the fraction of genotypes that require a single mutation to reach the new genotype network. This fraction increases - new genotype networks become easier to reach - as the penetrance of alternative phenotypes increases. The distance to a new genotype network decreases with increasing penetrance in all parameter combinations that we examined (Table 4; Spearman's *ρ*; *p *< 2.2 × 10^-16 ^in all cases). The same holds when we consider alternative phenotypes produced by the perturbation of two instead of just one gene in the initial condition (Additional file 3: Analysis S1).

**Table 4:** Genetic distance to a new genotype network decreases with increasing penetrance

*N*	*c*	*d*	Spearman's *ρ*	*p*-value	
8	0.4	0.25	-0.268	< 2.2 × 10^-16^	
		
		0.125	-0.211	< 2.2 × 10^-16^	
	
	0.3	0.25	-0.218	< 2.2 × 10^-16^	

16	0.35	0.25	-0.293	< 2.2 × 10^-16^	
	
	0.25	0.125	-0.263	< 2.2 × 10^-16^	
		
		0.25	-0.306	< 2.2 × 10^-16^	

20	0.3	0.25	-0.26	< 2.2 × 10^-16^	
	
	0.2	0.1	-0.269	< 2.2 × 10^-16^	
		
		0.25	-0.33	< 2.2 × 10^-16^	
		
		0.5	-0.4	< 2.2 × 10^-16^	
	
	0.1	0.25	-0.23	< 2.2 × 10^-16^	

Data for each parameter combination is based on 10^4 ^different circuits and on the non-parametric rank correlation coefficient Spearman's *ρ *[76].

We performed two additional complementary analyses, that we present in the Additional files. In the first analysis we found that genotypes that produce an alternative phenotype with high penetrance have more mutational paths towards the genotype network of that phenotype (Additional file 4: Analysis S2). In the second analysis we compared two kinds of sequences of mutations: those that increase the penetrance of an alternative phenotype $s_{\infty }^{new}$ and those that merely preserve the alternative phenotype $s_{\infty }^{new}$, regardless of its penetrance. We show that the former kind of mutations facilitates the arrival to $s_{\infty }^{new}$'s genotype network (Additional file 5: Analysis S3). Taken together, the observations in this section show that the genotype network of a new phenotype is closer and easier to reach from genotypes where a new phenotype has high penetrance.

### Plasticity accelerates the discovery of new genotype networks

Thus far, all our results support that plasticity facilitates discovery and stabilization of new phenotypes. We next asked more directly whether this is the case, by analyzing populations of circuits subject to repeated cycles of mutation and selection.

We started by establishing a population of *M *= 10^3 ^identical circuits. These circuits produce a phenotype $s_{\infty }^{native}$ from an initial state *s*_0_. We assigned individuals with this native phenotype a fitness (survival probability) *ω^native ^*< 1. Then, we chose a random gene activity phenotype $s_{\infty }^{new}$ as the target of an evolutionary search. We assigned individuals adopting $s_{\infty }^{new}$ a fitness of *ω^new ^*= 1. Starting from our initial population, we then carried out two parallel evolutionary simulations. In both we changed the population through repeated generations of replication, mutation and selection (see Methods).

In the first kind of simulation, we randomly perturbed each circuit's initial state every generation. We did so by perturbing each gene's initial activity state with a probability *α*. Then, we followed the circuit's gene activity dynamics, until the circuit attained its stable gene activity phenotype. We note that two circuits with the same genotype may produce different phenotypes because of these perturbations. We kept the rate of perturbation *α *low enough so that in most individuals the initial condition remained unperturbed. In the second, parallel 'control' simulation, we never perturbed the initial condition. Here, individuals needed to 'discover' the novel phenotype exclusively through mutation.

In both simulations we recorded two quantities. The first is the time (in generations) until the first individual in the population 'discovers' the genotype network of $s_{\infty }^{new}$. We call these times t_*,*plast *_and t_*,*control *_for the simulations with plasticity and the control simulations. The second is the time, from either t_*, *plast *_or t_*, *control*_, until at least one quarter of the population lies in the new genotype network. We call these times *t*_0.25, *plast *_and *t*_0.25, *control*_, respectively.

**Figure 5:**
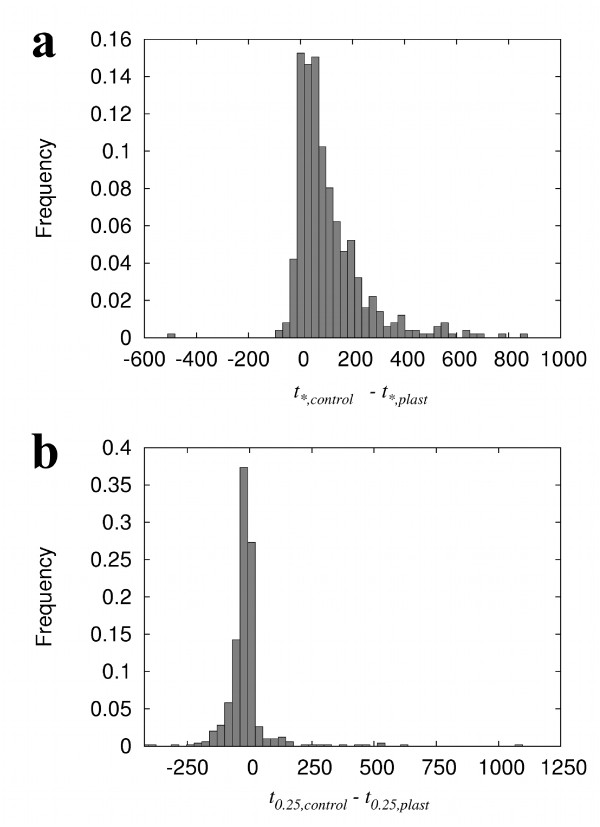
**Plasticity affects the speed of novel phenotype acquisition**. (a) Populations find a novel genotype network faster when plasticity is allowed. The symbol t_*, *plast *_refers to the number of generations that a population of circuits needs to discover a specific genotype network when we allow plasticity. The symbol t_*, *control *_refers to the same number, but for populations without plasticity. Wilcoxon signed-rank test; *p *< 2.2 × 10^-16^. (b) Plasticity slows the accumulation of individuals in the new genotype network. The symbol *t*_0.25,*plast *_stands for the number of generations that a population in which we allow plasticity needs to have at least 25 percent of its circuits in the new genotype network (after its discovery by a single individual); *t*_0.25,*control *_corresponds to the same number but without plastic phenotypes. Wilcoxon signed-rank test; *p *< 2.2 × 10^-16^. Both panels include data from 498 independent pairs of populations. Parameter values for both panels: *N *= 8, *c *≈ 0.4, *d *= 0.25, *μ *= 0.5, *α *= 0.05, *M *= 1000, *ω^native ^*= 0.5.

Figure 5a illustrates that populations in which we allow plasticity usually discover the new genotype network - a genetically determined novel phenotype - faster than control populations (t_*,*plast *_<t_*,*control*_). This observation holds after variation in values of *N*, *c *and *d*: with plasticity, the time to discovery of a new genotype network is significantly lower in all cases (Table 5; Wilcoxon signed-rank test). We also varied the rate of perturbation per gene in *s*_0_, the fitness of the native phenotype *ω^native^*, and the population size *M*. Nearly all such variations yielded a significantly shorter discovery time when we allowed phenotypic plasticity (Table 6; Wilcoxon signed-rank test). In sum, plasticity robustly accelerated the discovery of new, genetically stable phenotypes.

**Table 5:** Plasticity accelerates the discovery of a new optimal genotype network

*N*	*c*	*d*	Sample size	Mean t_*, *control*_	Mean t_*, *plast*_	*p*-value
8	0.4	0.125	498	164.93	54.45	< 2.2 × 10^-16^
		
		0.25	498	160.3	52.86	< 2.2 × 10^-16^
		
		0.5	497	167.58	46.95	< 2.2 × 10^-16^
	
	0.3	0.125	495	104.92	42.82	< 2.2 × 10^-16^
		
		0.25	498	103.84	38.03	< 2.2 × 10^-16^
		
		0.5	498	136.33	40.53	< 2.2 × 10^-16^

16	0.25	0.125	420	2744.8	2319.81	0.00059
		
		0.25	435	2792.44	2299.51	3.9 × 10^-5^
		
		0.5	421	2832.02	2173.54	2.2 × 10^-7^
	
	0.2	0.125	464	2510.68	1700.95	1.4 × 10^-11^
		
		0.25	468	2483.48	1867.23	1.2 × 10^-6^
		
		0.5	473	2400.1	1758.22	1.4 × 10^-7^

20	0.2	0.25	154	3961.17	3303.32	0.006869

The number of generations that a population takes to 'discover' a circuit in a new genotype network is significantly lower when we allow plasticity (t_*, *plast *_<t_*, *control*_), according to a Wilcoxon signed-rank test.

The value of *d *is that of the old genotype network. We analyzed 500 pairs of evolving populations for each combination of *N*, *c *and *d*. We discarded population pairs in which any of the populations had not reached the new genotype network by the end of the simulation (*t *= 10^4^). Thus, our actual sample size was lower than 500 populations. The probability *α *ofgene-activity perturbation in *s*_0 _equaled 0.05 per gene when *N *= 8, 0.025 when *N *= 16, and 0.02 when *N *= 20. Population size *M *= 1000; *μ *= 0.5; *ω^native ^*= 0.5.

**Table 6:** For nearly all combinations of parameter values, plasticity accelerates the discovery of a new optimal genotype network

*N*	*α*	***ω***^***native***^	*M*	Sample size	Mean t_*, *control*_	Mean t_*, *plast*_	*p*-value
8	0.02	0.5	1000	498	160.3	55.1	< 2.2 × 10^-16^
				
	0.08			498	160.3	53.98	< 2.2 × 10^-16^
				
	0.01			498	160.3	53.49	< 2.2 × 10^-16^
			
	0.05		200	498	482.27	129.2	< 2.2 × 10^-16^
			
			10000	498	63.06	25.14	< 2.2 × 10^-16^
		
		0.2	1000	498	53.57	29.74	< 2.2 × 10^-16^
				
		0.8		498	235.86	70.17	< 2.2 × 10^-16^
				
		0.95		498	251.77	79.7	< 2.2 × 10^-16^

16	0.01	0.5	1000	467	2476.75	1858.21	4.2 × 10^-6^
			
	0.05			462	2437.04	1886.83	0.0001328
			
	0.025		200	248	3381.98	3389.867	0.778
			
			10000	500	548.65	392.58	1.5 × 10^-14^
		
		0.2	1000	500	225.74	187.78	5.4 × 10^-7^
				
		0.8		283*^a^*	4172.47	3494.55	0.00204

The number of generations that a population takes to 'discover' a circuit in a new genotype network is significantly lower when we allow plasticity (t_*, *plast *_<t_*, *control*_), according to a Wilcoxon signed-rank test.

The value of *d *is that of the old genotype network. We analyzed 500 pairs of evolving populations for each combination of *N*, *c *and *d*. We discarded population pairs in which any of the populations had not reached the new genotype network by the end of the simulation (*t *= 10^4^). Thus, our actual sample size was lower than 500 populations. The remaining parameter values are as follows: *d *= 0.25; *μ *= 0.5. *c *≈ 0.4 when *N *= 8 and it equals 0.2 when *N *= 16.

*^a ^*We analyzed 1500 pairs of evolving populations for the simulations that we report in this row, but the populations found the new genotype network only in 283 cases.

In contrast to these observations, the time from the discovery of a new genotype network until 25 percent of a population occupied this genotype network was longer for populations in which we allowed plasticity (*t*_0.25,*control *_<*t*_0.25,*plast*_; Figure 5b). This difference, albeit small, is statistically significant for all combinations of values for *N*, *c *and *d *that we tested (Table 7). Thus, plasticity can slow the rate at which individuals with a genetically determined new phenotype increase in frequency. In sum, the shorter discovery time of a new genotype network associated with phenotypic plasticity is followed by a slower transition into this new network. The small plasticity-dependent delay that we observe in our work coincides with predictions of a previously published theoretical study [29]. In that study, the delay arises because of a decreased selection differential associated with alleles that have conditional fitness effects.

**Table 7:** Plasticity slows down the frequency increase of circuits in a new optimal genotype network.

*N*	*c*	*d*	Sample size	Mean *t*_0.25,*control*_	Mean *t*_0.25,*plast*_	*p*-value
8	0.4	0.125	498	27.55	46.72	< 2.2 × 10^-16^
		
		0.25	498	30.69	45.91	< 2.2 × 10^-16^
		
		0.5	497	28.01	45.63	< 2.2 × 10^-16^
	
	0.3	0.125	495	18.8	26.49	< 2.2 × 10^-16^
		
		0.25	498	19.25	25.86	< 2.2 × 10^-16^
		
		0.5	498	26.44	25.99	< 2.2 × 10^-16^

16	0.25	0.125	413	41.61	106.96	6.9 × 10^-14^
		
		0.25	431	35.66	138.98	< 2.2 × 10^-16^
		
		0.5	418	49.76	162.98	< 2.2 × 10^-16^
	
	0.2	0.125	462	43.4	143.45	< 2.2 × 10^-16^
		
		0.25	466	36.48	133.72	< 2.2 × 10^-16^
		
		0.5	471	42.01	120.97	< 2.2 × 10^-16^

20	0.2	0.25	152	35.45	103.8	4.4 × 10^-10^

The number of generations that a population needs to increase the fraction of its circuits in the new genotype network to 25 percent is significantly higher with plasticity, i.e., *t*_0.25,*plast *_>*t*_0.25,*control *_according to a Wilcoxon signed-rank test.

The value of *d *is that of the old genotype network. We analyzed 500 pairs of evolving populations for each combination of *N*, *c *and *d*. We discarded population pairs in which any of the populations never had 25% of its circuits in the new genotype network by the end of the simulation (*t *= 10^4^). Thus, our actual sample size was lower than 500 populations. The probability *α *of gene-activity perturbation in *s*_0 _equaled 0.05 per gene when *N *= 8, 0.025 when *N *= 16, and 0.02 when *N *= 20. Population size *M *= 1000; *μ *= 0.5; *ω^native ^*= 0.5.

## Discussion

**Figure 6:**
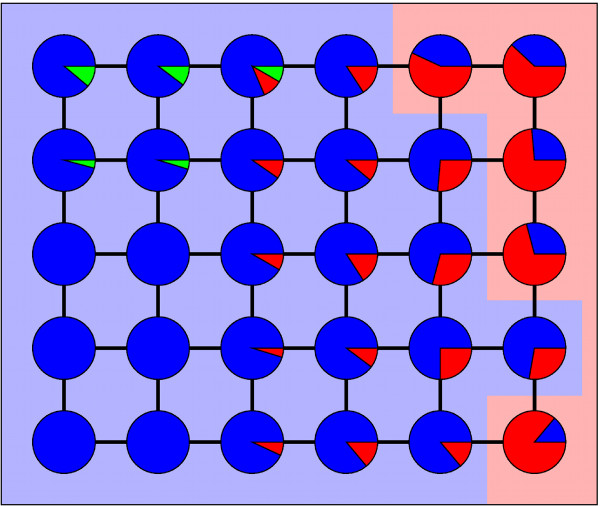
**The organization of gene circuit genotype space favors plasticity-mediated adaptive evolution**. Shapes and colors have the same meanings in the diagram as in Figure 1. Starting from the blue genotype network, it is easier to find a genotype that produces a red alternative phenotype than finding the red genotype network. Many genotypes that produce the red alternative phenotype have direct mutational access to the red genotype network. Such genotypes are neighbors of other genotypes that produce the same alternative phenotype. Among genotypes that produce the red alternative phenotype, a high penetrance is associated to easier access to the red genotype network.

The model of gene regulatory circuits we use here is a coarse, abstract representation of the real complexity of such circuits. This is its main limitation and, at the same time, its strength. It allows us to analyze millions of circuits, their native phenotypes, and any alternative phenotypes they might adopt. In other words, it allows us to determine how gene expression phenotypes are organized in circuit genotype space. This organization has several properties that facilitate the discovery of a new phenotype through plasticity and its genetic stabilization (Figure 6). First, many circuits show plastic phenotypes, which helps them explore many more novel phenotypes than mutation alone could. Second, many circuits that can produce a new phenotype *P_new _*through plasticity are neighbors of the genotype network of *P_new_*. They thus allow mutational access to a genotype whose native phenotype is *P_new_*. Third, similar genotypes on the same genotype network often produce the same alternative phenotypes. Fourth, the higher the penetrance of a new phenotype *P_new _*in a genotype *G *is, the easier it is to reach *P_new_*'s genotype network from *G*. From these observations emerges an important role for plasticity in the discovery and stabilization of novel phenotypes (Figure 1): Genotypes that produce a new phenotype through plasticity (i.e. after non-genetic perturbations) may frequently be intermediates in evolutionary paths towards genotypes where that phenotype is the 'native' phenotype.

Evolutionary simulations show that plasticity can indeed accelerate evolutionary adaptation. In these simulations we determined the time a population needs to 'discover' the genotype network of an arbitrary novel and adaptive phenotype. This time is significantly lower when phenotypes are plastic than when they are not.

At the same time, plasticity causes a small reduction in the rate at which the frequency of circuits in a new genotype network increases. The reason is straightforward. In the absence of plasticity, genotypes in the new genotype network always produce the new, best adapted phenotype. With plasticity, the same genotypes occasionally produce other, suboptimal phenotypes: These genotypes are thus overall less well adapted than in the absence of plasticity, which slows their ascent in the population. The resulting delay is similar to that predicted by previous theoretical work [22,29,73,74] that showed smaller selection differentials in populations with plasticity. These smaller differentials can result from either the different fitness effects of the distinct phenotypes that a genotype may produce [22,74], or from alleles that are visible to selection only under certain environmental or genetic conditions [29,73]. At least in our study system, this delay is insufficient to cancel out the acceleration in finding a new genotype network. Plasticity accelerates the discovery of the new genotype network to a much greater extent than it slows down the 'conquest' of this network by a population (Tables 5, 7).

In our system, the positive role of plasticity in the origin and stabilization of new phenotypes is closely tied to the existence of large genotype networks. Such networks allow access to very distant regions in genotype space, while preserving an existing native phenotype. And because the spectrum of alternative phenotypes in these regions is not the same, genotype networks facilitate the exploration of many novel phenotypes through plasticity.

Early studies, albeit based on an unrealistic genotype-phenotype map, suggested that plasticity could improve the efficiency of an evolutionary search by smoothing fitness landscapes [75]. Ancel and Fontana asked related questions with a more realistic genotype-phenotype map: the folding of RNA molecules [22]. These authors showed that plasticity does not accelerate adaptive evolution in RNA structures. The reason is that RNA molecules lack the first of our four properties: It is not much easier to discover a novel alternative phenotype than to discover this phenotype's genotype network. This occurs because finding an RNA genotype capable of producing two specific secondary structure phenotypes severely constrains the allowed base-pairings in each RNA structure [22].

In contrast to the RNA system, plasticity has a positive role for evolutionary adaptation in our regulatory circuits. Thus, the role of plasticity may depend on the level of biological organization one focuses on. We note that much of the empirical evidence supporting plasticity's importance for adaptation comes from morphological traits, such as head size in snakes, and developmental decisions, such as those involving spore formation in bacteria [30-35,42,46]. In this regard, it is significant that gene regulatory circuits are central to shape these kinds of traits. It may thus not be a coincidence that plasticity can facilitate evolutionary transitions in these traits. Our modeling results suggest that future experimental analyses will produce observations similar to existing experiments and similar to our observations wherever regulatory circuits are involved in adaptation.

Transcriptional regulation circuits, while important, are not the only systems in which change can lead to novel traits that confer a fitness advantage. Changes in mechanical, biochemical or geometrical cell and tissue properties, in cell-cell signaling, in hormonal communication between distant parts of the organism, or even in behavioral traits can lead to new, potentially adaptive, phenotypes. Whether plasticity facilitates adaptive evolution in all these traits and systems is unknown. To answer this question will require experimental studies of evolution in individual, mechanistically well-understood systems associated with innovation, combined with computational work like ours that reveals generic principles of evolution for a system class. In addition, the changes that constitute an adaptive novelty frequently span several levels of biological organization and multiple kinds of systems. New theoretical and empirical approaches are needed to ask how such different levels of biological organization interact in producing phenotypic variation. Such approaches will be important to understand the evolutionary origin of complex adaptations and the role of plasticity beyond individual case studies.

## Conclusions

Our results predict that phenotypic plasticity will have an important role in adaptive evolution that involves changes in gene activity. The fundamental reason is that genotypes that produce occasionally a beneficial phenotype (and thus have a selective advantage) give more easily rise to genotypes where that same phenotype is more strongly genetically determined. New adaptive phenotypes may frequently arise first as alternative phenotypes, induced by non-genetic perturbations, and then be genetically stabilized by selection. Our work thus suggests a widespread relationship between phenotypic plasticity and adaptive evolution in gene activity phenotypes.

## Methods

Throughout this work, we chose new non-zero entries in a circuit's interaction matrix from a normal distribution with mean zero and standard deviation one. Also throughout this work, we disregarded the phenotypes of circuits that attained no stable fixed-point activity pattern, as in previous studies [11].

### Sampling of random genotypes in a genotype network

To sample genotypes, we first used previously established procedures to identify a circuit genotype that produces a certain gene activity phenotype $s_{\infty }^{native}$ from an initial condition s_0 _[7]. Then, we imposed 10^4 ^random mutations that preserve the production of $s_{\infty }^{native}$ from *s*_0_, to erase any trace of the initial genotype. Each mutation was picked at random from many possible alternatives (see 'Phenotypes accessible through mutation' below). The genotype obtained in this manner is the first in our sample. To sample additional genotypes in the same genotype network, we subjected the genotype to a series of 5*m*_+ _mutations that we required to preserve $s_{\infty }^{native}$. Here, *m*_+ _stands for the maximum number of interactions allowed in a circuit. This procedure allowed us to avoid correlations between consecutively sampled genotypes.

### Phenotypes accessible through perturbations in the initial condition *s*_0_

For a circuit genotype that attains $s_{\infty }^{native}$ when its developmental dynamics start from *s*_0_, we determined the phenotype of the *N *possible single gene-activity modifications of *s*_0_. Wherever we explored the effects of double gene perturbations of the initial condition, we followed the same procedure, but for all *N*(*N *- 1)/2 possible two-gene perturbations.

### Phenotypes accessible through mutation

To generate all neighbors of a circuit, we examined each entry *a_ij _*in the matrix **A **of the circuit, and distinguished the following cases: if *a_ij _*≠ 0 we determined the phenotype of two mutants, one in which the circuit loses an interaction (*a_ij _*= 0), and one in which the value of *a_ij _*changes but we force its sign to remain unaltered; if *a_ij _*= 0 we obtain the phenotype of two mutants, one in which *a_ij _*> 0, and one in which *a_ij _*< 0. Whenever we required a value of *a_ij _*to be either smaller or greater than zero, we forced its sign as needed. To find the phenotype of a mutant circuit we followed the developmental dynamics (Eq. 1) using the unperturbed *s*_0 _activity pattern as initial condition. We only determined the phenotype of mutants whose number of interactions was equal or smaller than a pre-specified value *m *and equal or greater than *m *- 5. This allowed us to keep interaction density *c *in a narrow interval, in order to explore the effect of variations on *c*.

### Overlap between *P_μ _*and $P_{s_{0}}$

We used the index $C=\frac{\left|P_{\mu }\cap P_{s_{0}}\right|}{\min\left(\left|P_{\mu }|,|P_{s_{0}}\right|\right)}$ to assess the similarity between the set of phenotypes that a genotype *G *produces after single mutations (*P_μ_*) and the set of phenotypes that *G *produces after perturbations in the initial condition ($P_{s_{0}}$). We obtained values of *C *for each of 10^4 ^genotypes in a pre-determined genotype network. Throughout this sampling, we built two lists of phenotypes, *L_μ _*and $L_{s_{0}}$. We kept in either *L_μ _*or $L_{s_{0}}$ the phenotypes that genotypes in the sample could access through either mutation or perturbations in *s*_0_, respectively. Then, we determined for each genotype a second index *C^rand^*, that measures the similarity between *P_μ _*and $P_{s_{0}}$ expected by chance alone. We calculated *C^rand ^*as we calculated *C*, but from randomized phenotype sets. specifically, we randomized a genotype's *P_μ _*($P_{s_{0}}$) by replacing each phenotype in this set by a gene activity pattern picked at random from *L_μ _*($L_{s_{0}}$). We did not allow any phenotype to appear more than once in any one randomized set. Among the 10^4 ^genotypes that we sampled, we discarded those in which either *P_μ _*or $P_{s_{0}}$ was empty.

### Shortest mutational distance to a new genotype network

We picked a genotype *G *at random from the genotype network of a pre-specified phenotype $s_{\infty }^{native}$. We selected at random one of *G*'s alternative phenotypes, and called it $s_{\infty }^{new}$. We created 10^3 ^copies of *G*, and changed each copy's regulatory interactions in a sequence of mutational steps. None of the steps was allowed to leave $s_{\infty }^{native}$'s genotype network. We stopped the sequence as soon as one of the circuits was a neighbor of $s_{\infty }^{new}$'s genotype network. This procedure allowed us to estimate an upper-bound for the minimum number of mutations required to reach $s_{\infty }^{new}$'s genotype network from *G*.

### Evolutionary simulations

We established a population of *M *= 10^3 ^identical circuits. Such circuits produce a phenotype $s_{\infty }^{native}$ from an initial state *s*_0_. We also picked a gene activity pattern where each gene's activity state was picked at random. This activity pattern represents the new optimal phenotype $s_{\infty }^{new}$ and it is different from the native phenotype $s_{\infty }^{native}$. We let the population evolve through repeated cycles ('generations') of mutation and selection implemented as follows.

To assess a circuit's phenotype and fitness, we first assigned an initial condition to the circuit by changing the activity state of each gene in the unperturbed initial condition *s*_0 _with a probability *α*. We used this perturbed initial condition to start the gene activity dynamics dictated by the circuit's matrix **A **(Eq. 1). The resulting stable (fixed-point) activity pattern *s_∞ _*is the circuit's phenotype. We disregarded circuits that did not produce a fixed-point equilibrium state. If a circuit's phenotype s*_∞ _*was equal to $s_{\infty }^{new}$, then we assigned to it a fitness *ω *= *ω^new ^*= 1. Otherwise, we set its fitness *ω *equal to the maximum of either (1 - *y_new_/*N)^5 ^or *ω^native^*(1 - *y_native_/*N)^5^, where *y_new _*equals the number of gene activity differences between the phenotype *s_∞ _*and $s_{\infty }^{new}$, and *y_native _*equals the number of gene activity differences between *s_∞ _*and $s_{\infty }^{native}$. This procedure ensures a steep decrease in fitness for any deviations from gene expression states $s_{\infty }^{new}$ or $s_{\infty }^{native}$.

We chose the value of *α *so that no gene activity was perturbed in the majority of circuits. Unless indicated otherwise, *α *= 0.02 when *N *= 20, *α *= 0.025 when *N *= 16 and *α *= 0.05 when *N *= 8. To study evolutionary dynamics without plasticity, we set *α *equal to zero in control populations. In these populations, circuits always start their developmental dynamics from *s*_0_.

At each generation, we sampled individuals from the current population with replacement. For every sampled genotype *G*, we subjected with probability *μ *a copy of *G *to mutation, thus picking one of its (mutational) neighbors at random (see 'Phenotypes accessible through mutation' above). We allowed such a new circuit in the new generation with a probability equal to its fitness. In other words, we considered fitness as survival probability. We continued sampling until *M *new individuals constituted the population for the next generation. We stopped evolution after either at least one quarter of the population resided in the new genotype network, or after 10^4 ^generations, whichever came first. For all simulations, *ω^native ^*= 0.5, *M *= 10^3^, *μ *= 0.5, unless indicated otherwise.

## Authors' contributions

CE-S designed most of the analyses, wrote program scripts, carried out the analyses, interpreted the results and drafted the manuscript. OCM contributed program codes, designed some of the analyses, and drafted the manuscript. AW designed some of the analyses, interpreted the results and drafted the manuscript. All authors read and approved the final manuscript.

## Supplementary Material

Additional file 1 **Figure S1**. Gene circuits exploring a genotype network find genotypes with new alternative phenotypes faster than they find new genotype networks, for different parameter combinations. Click here for file

Additional file 2 **Figure S2**. Mutations and perturbations in the initial condition *s*_0 _produce the same phenotypes more often than expected by chance. Click here for file

Additional file 3 **Analysis S1**. Genetic distance to a genotype network decreases with increasing phenotypic penetrance, for alternative phenotypes produced after two-gene perturbations in the initial condition. Click here for file

Additional file 4 **Analysis S2**. High penetrance increases the number of mutational paths to a new genotype network. Click here for file

Additional file 5 **Analysis S3**. Mutations that increase in penetrance facilitate access to a new genotype network. Click here for file
