# Supplementary material for: Phenotypic plasticity can facilitate adaptive evolution in gene regulatory circuits
Source: BMC Evol Biol. 2011 Jan 6;11:5. doi: 10.1186/1471-2148-11-5 (PMC3024936; doi:10.1186/1471-2148-11-5)
Supplement: Additional file 1 — Figure S1. Gene circuits exploring a genotype network find genotypes with new alternative phenotypes faster than they find new genotype networks, for different parameter combinations. [file 1471-2148-11-5-S1.PDF]

Additional file 1

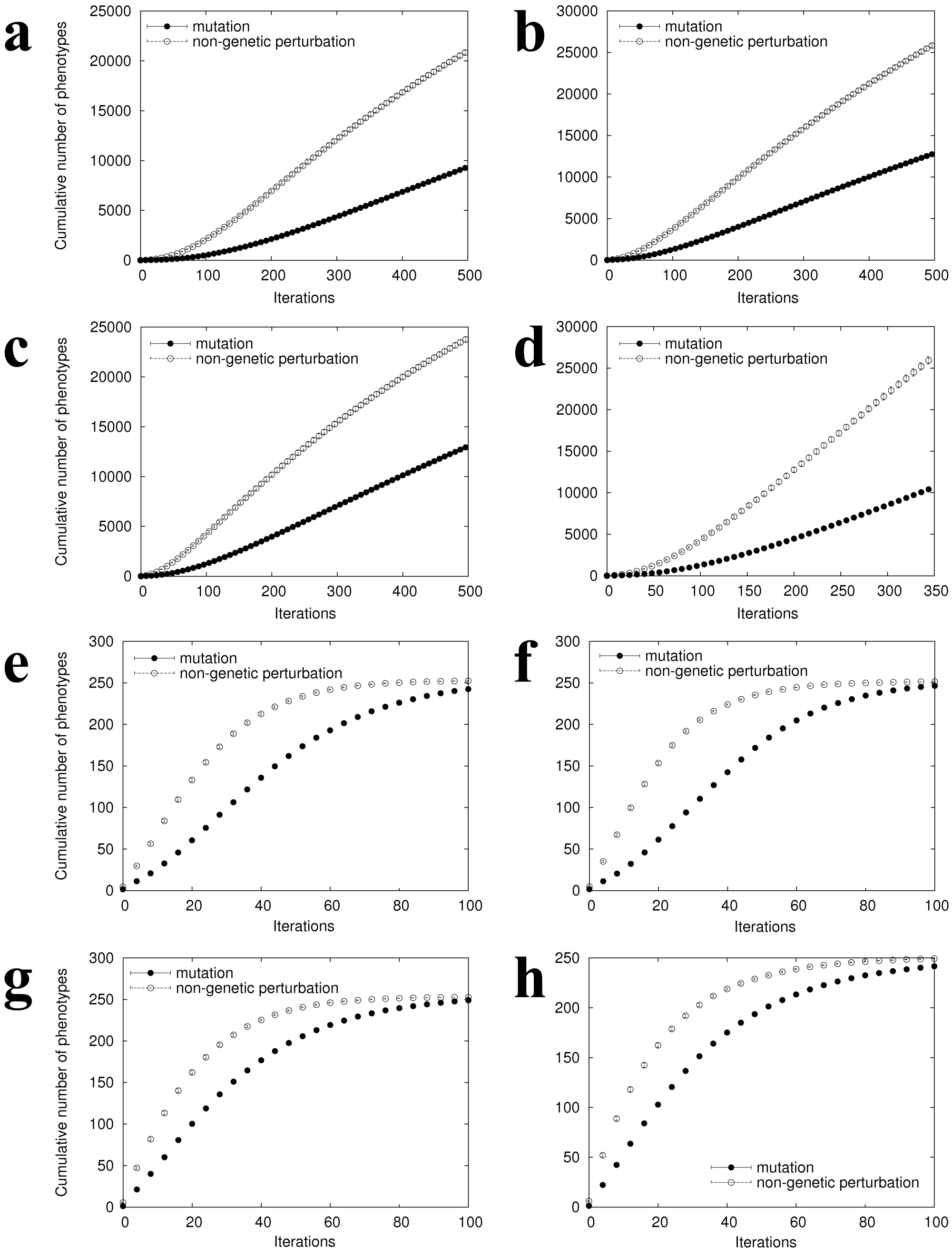

Figure S1. Gene circuits exploring a genotype network find genotypes with new alternative phenotypes faster than they find new genotype networks. Each panel shows mean values for 200 independent samples, each of size $M = 5 \times 10^3$ circuits. a) $N = 16$, $c \approx 0.35$, $d = 0.5$. b) $N = 16$, $c \approx 0.25$, $d = 0.125$. c) $N = 16$, $c \approx 0.25$, $d = 0.5$. d) $N = 20$, $c \approx 0.2$, $d = 0.25$. e) $N = 8$, $c \approx 0.4$, $d = 0.125$. f) $N = 8$, $c \approx 0.4$, $d = 0.5$. g) $N = 8$, $c \approx 0.3$, $d = 0.125$. h) $N = 8$, $c \approx 0.3$, $d = 0.5$.
