# Supplementary material for: Phenotypic plasticity can facilitate adaptive evolution in gene regulatory circuits
Source: BMC Evol Biol. 2011 Jan 6;11:5. doi: 10.1186/1471-2148-11-5 (PMC3024936; doi:10.1186/1471-2148-11-5)
Supplement: Additional file 2 — Figure S2. Mutations and perturbations in the initial condition s0 produce the same phenotypes more often than expected by chance. [file 1471-2148-11-5-S2.PDF]

Additional file 2

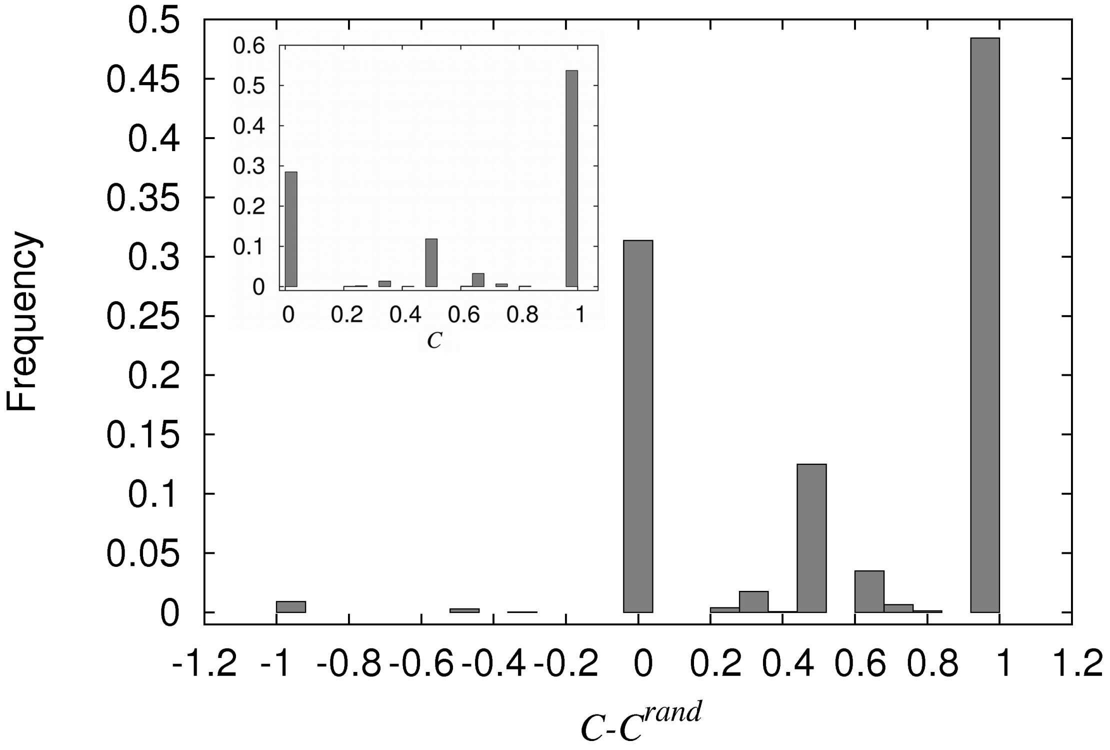

Figure S2. Mutations and perturbations in the initial condition s_0 produce the same phenotypes more often than expected by chance. The sample included 10^4 genotypes in a genotype network, but genotypes that did not produce new phenotypes under either kind of perturbation were discarded. In this plot we show results for circuits with $N = 20$ genes, an interaction density $c \approx 0.2$, and a fraction d of gene activity differences between the unperturbed initial condition s_0 and the native phenotype s_∞^{native} equal to 0.1. C is significantly higher than C^{rand} (Wilcoxon signed-rank test; $p < 2.2 \times 10^{-16}$). The inset shows the distribution of the values of C for the same sample.
