# Supplementary material for: Phenotypic plasticity can facilitate adaptive evolution in gene regulatory circuits
Source: BMC Evol Biol. 2011 Jan 6;11:5. doi: 10.1186/1471-2148-11-5 (PMC3024936; doi:10.1186/1471-2148-11-5)
Supplement: Additional file 3 — Analysis S1. Genetic distance to a genotype network decreases with increasing phenotypic penetrance, for alternative phenotypes produced after two-gene perturbations in the initial condition. [file 1471-2148-11-5-S3.PDF]

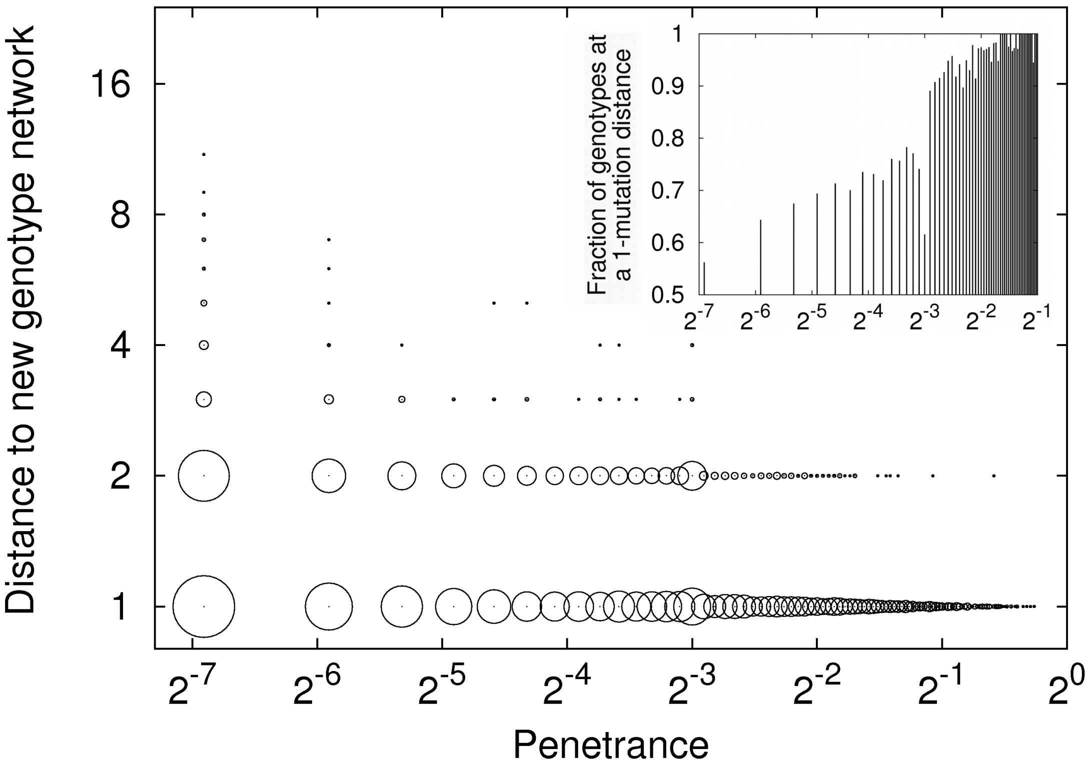

Figure S3. Genetic distance to a genotype network decreases with increasing phenotypic penetrance, for alternative phenotypes produced after two-gene perturbations in the initial condition. The horizontal axis corresponds to the fraction of different two-gene perturbations that produce a phenotype s_{∞}^{new}. The vertical axis corresponds to the number of mutations required to reach the genotype network of s_{∞}^{new}. A circle's area is proportional to the number of data points for a particular penetrance/mutational distance category. The figure includes 10^4 data points for circuits with $N = 16$, $c \approx 0.35$, and $d = 0.25$. Spearman's $\rho = -0.31$; $p < 2.2 \times 10^{-16}$.

Table S1. Genetic distance to a genotype network is negatively correlated to a phenotype's penetrance. For this table, we considered alternative phenotypes that a circuit produces after two-gene perturbations in the initial condition. We assayed 10^4 different genotypes for each parameter combination.

N	c	d	Spearman's ρ	p-value
	0.4	0.25	-0.229	$< 2.2 \times 10^{-16}$
8		0.125	-0.186	$< 2.2 \times 10^{-16}$
	0.3	0.25	-0.138	$< 2.2 \times 10^{-16}$
	0.35	0.25	-0.31	$< 2.2 \times 10^{-16}$
16	0.25	0.125	-0.28	$< 2.2 \times 10^{-16}$
		0.25	-0.295	$< 2.2 \times 10^{-16}$
	0.3	0.25	-0.303	$< 2.2 \times 10^{-16}$
		0.1	-0.291	$< 2.2 \times 10^{-16}$
20	0.2	0.25	-0.319	$< 2.2 \times 10^{-16}$
		0.5	-0.359	$< 2.2 \times 10^{-16}$
	0.1	0.25	-0.303	$< 2.2 \times 10^{-16}$
