# Supplementary material for: Phenotypic plasticity can facilitate adaptive evolution in gene regulatory circuits
Source: BMC Evol Biol. 2011 Jan 6;11:5. doi: 10.1186/1471-2148-11-5 (PMC3024936; doi:10.1186/1471-2148-11-5)
Supplement: Additional file 4 — Analysis S2. High penetrance increases the number of mutational paths to a new genotype network. [file 1471-2148-11-5-S4.PDF]

Additional file 4 — Analysis S2

We analyzed randomly chosen genotypes G that (i) can produce an alternative phenotype, and (ii) are two mutations away from this phenotype's genotype network. We focused on such genotypes, because genotypes further away from the new genotype network show very little variation in penetrance (figure 4b). We ask whether genotypes where an alternative phenotype is more penetrant can follow a a higher number of shortest mutational paths to the phenotype's genotype network (see figure below).

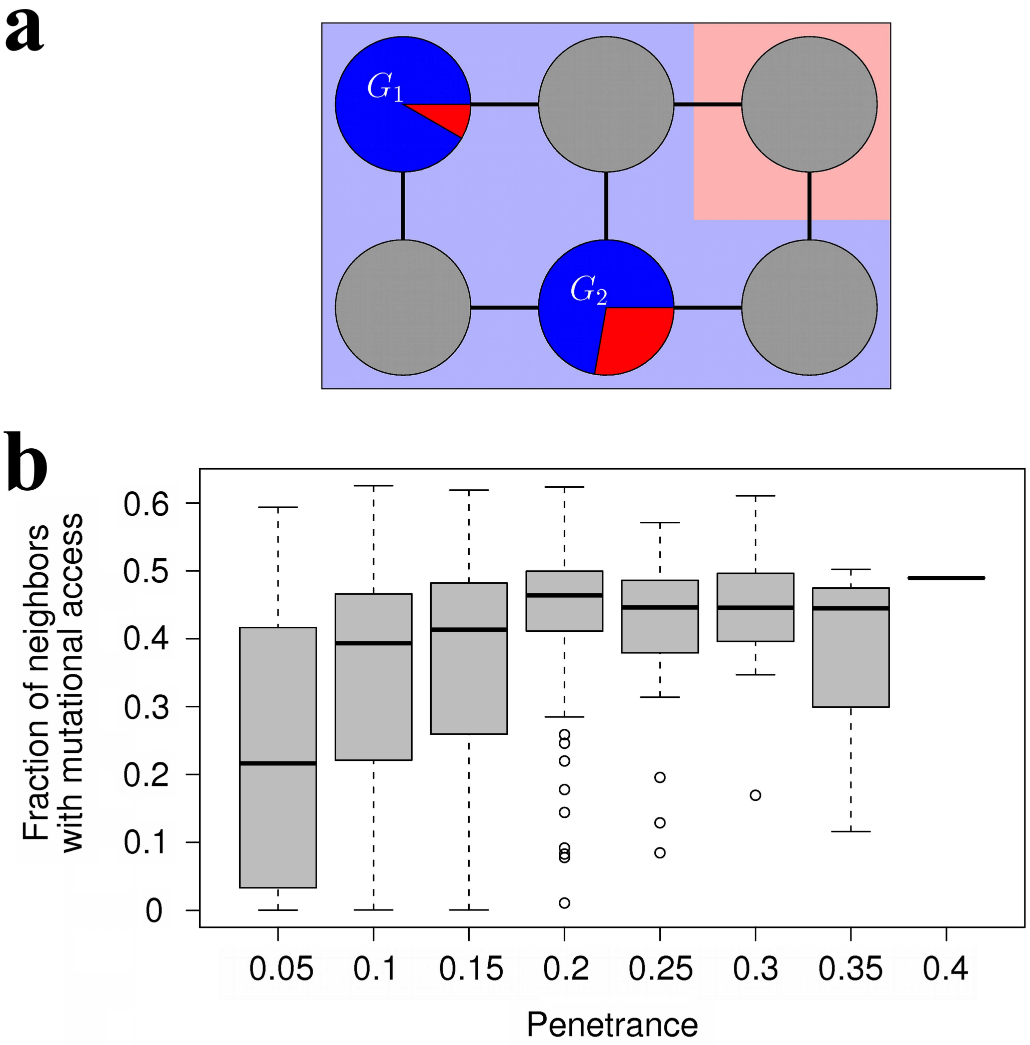

Figure S4. High penetrance increases the number of mutational paths to a new genotype network. (a)The principle of our analysis. In this panel, two hypothetical genotypes G_1 and G_2 differ in the penetrance of an alternative phenotype s_∞^{new} (red sectors). Both genotypes require two mutations to reach the genotype network of s_∞^{new}. G_1, in which s_∞^{new} has a low penetrance, has a single neighbor with direct mutational access to s_∞^{new}'s genotype network. In contrast, G_2, in which s_∞^{new} has a higher penetrance, has two neighbors with direct mutational access to s_∞^{new}'s genotype network. Thus, in this hypothetical example, despite G_1 and G_2 being at the same distance of the new genotype network, a high penetrance entails a higher probability of reaching the new genotype network. Our analysis asks whether the illustration's scenario applies to genotypes of gene circuits. (b) A high penetrance is positively associated with a high fraction of neighbors of G that are themselves neighbors of (have mutational access to) the new genotype network. The data is based on 9803 genotypes, for circuits of $N = 20$ genes, an interaction density $c \approx 0.2$ and a distance between s_0 and s_∞^{native} $d = 0.25$. The thick line in the middle of each rectangle represents the median of the distribution. Each rectangle shows the interquartile range (i.e. the range from the first to the third quartile). Whiskers show the smallest (largest) value within 1.58 times the interquartile range from the first (third) quartile. Points outside whiskers are outliers. Penetrance and the fraction of neighbors with mutational access are positively associated (Spearman's $\rho = 0.215$; $p < 2.2 \times 10^{-16}$).

Table S2. High penetrance increases the number of mutational paths to a new genotype network. We analyzed randomly chosen genotypes G that (i) can produce an alternative phenotype, and (ii) are two mutations away from this phenotype's genotype network. The data shows that high penetrance is positively associated with a high fraction of neighbors of G that are themselves neighbors of (have mutational access to) the new genotype network.

N	c	d	Sample size	Spearman's ρ	p-value
8	0.4	0.25	9128	0.157	$< 2.2 \times 10^{-16}$
		0.125	8364	0.082	2.4×10^{-14}
	0.3	0.25	7529	0.041	1.7×10^{-4}
16	0.35	0.25	9917	0.234	$< 2.2 \times 10^{-16}$
	0.25	0.125	9466	0.136	$< 2.2 \times 10^{-16}$
		0.25	9731	0.207	$< 2.2 \times 10^{-16}$
20	0.3	0.25	9957	0.238	$< 2.2 \times 10^{-16}$
	0.2	0.1	9568	0.13	$< 2.2 \times 10^{-16}$
		0.25	9803	0.215	$< 2.2 \times 10^{-16}$
		0.5	9929	0.285	$< 2.2 \times 10^{-16}$
	0.1	0.25	8539	0.05	2.1×10^{-6}
