# Supplementary material for: Phenotypic plasticity can facilitate adaptive evolution in gene regulatory circuits
Source: BMC Evol Biol. 2011 Jan 6;11:5. doi: 10.1186/1471-2148-11-5 (PMC3024936; doi:10.1186/1471-2148-11-5)
Supplement: Additional file 5 — Analysis S3. Mutations that increase in penetrance facilitate access to a new genotype network. [file 1471-2148-11-5-S5.PDF]

Additional file 5 — Analysis S3

We examined sequences of two kinds of mutations: The first kind are mutations towards neighboring genotypes where an alternative phenotype s_{∞}^{new} is most penetrant among all neighboring genotypes. The second kind are mutations that preserve s_{∞}^{new} as an alternative phenotype, irrespective of its penetrance (panel a in figure below).

Given an initial genotype, we generated 10^3 sequences of mutations according to the following procedures for each mutation in each sequence. (i) We first determined the genotypes that differed from the current genotype by a single mutation, that belonged to the same genotype network as the original genotype, and that could produce s_{∞}^{new}. Within this set of genotypes, we kept only those where s_{∞}^{new} had the highest penetrance, and chose a genotype at random among them. This genotype became the starting genotype for the next mutation in the sequence. The sequence of mutations concluded either when the current genotype was a neighbor of the genotype network of s_{∞}^{new}, or after the length of the sequence exceeded that of the shortest previous sequence that had reached the new genotype network. If no previous sequence had reached the new genotype network, we stopped the series after twelve mutations. (This number was sufficiently high to exclude observational artefacts, because we always found a shorter sequence of mutations to the new genotype network.) We report the fraction of sequences of mutations, among the 10^3 that we tried, that reached the new genotype network in the smallest number of mutations. (ii) We followed the same procedure as in (i) but without filtering genotypes according to penetrance. Overall, we determined such mutational sequences for each of 5×10^3 initial genotypes.

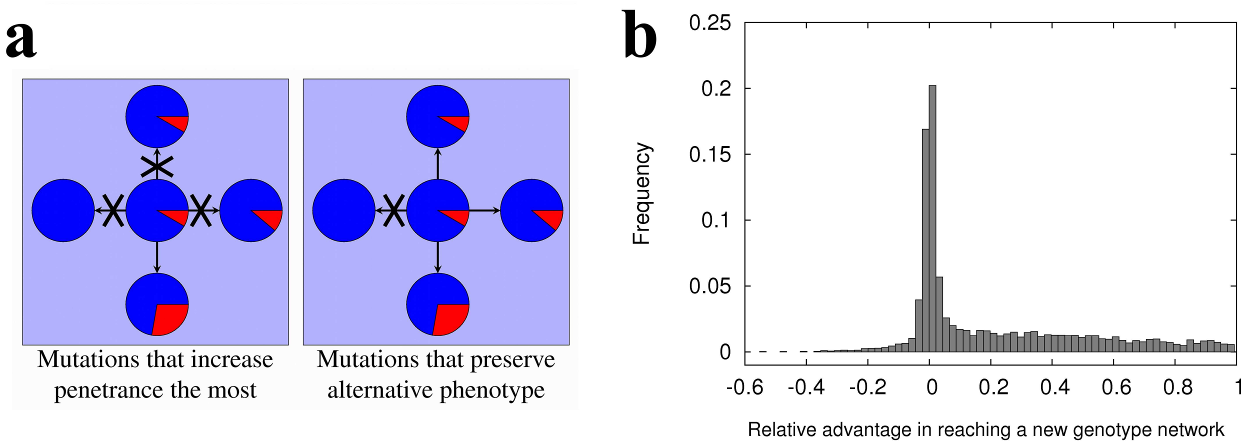

Figure S5. An increase in penetrance facilitates access to a new genotype network. (a) The principle behind our analysis. We examined sequences of two kinds of mutations: The first kind are mutations towards neighboring genotypes where an alternative phenotype s_{∞}^{new} is most penetrant among all neighboring genotypes (left). The second kind are mutations that preserve s_{∞}^{new} as an alternative phenotype, irrespective of its penetrance (right). Circles represent genotypes, and the size of red sectors indicates the penetrance of s_{∞}^{new}. Arrows between circles represent mutations that transform the central genotype into one of the other genotypes. Crossed-out arrows are mutations that would not be allowed in a sequence of mutations. We started from randomly chosen genotypes that could produce a given alternative phenotype s_{∞}^{new}, but that were not themselves neighbors of this phenotype's genotype network.(b) An increase in penetrance facilitates access to new genotype networks. The x-axis shows the advantage that mutations which increase penetrance maximally confer in reaching a new genotype network. We define this advantage V as the fraction of shortest mutation sequences that reach s_{∞}^{new}'s genotype network for mutations that increase penetrance maximally minus the same fraction for mutations that preserve s_{∞}^{new} irrespective of penetrance. The y-axis shows the frequency with which an advantage V is found in our sample of 5×10^3 initial genotypes. The data show that mutations that increase penetrance maximally facilitate access to a new genotype network. Wilcoxon-signed rank test; $p < 2.2 \times 10^{-16}$. For this figure we consider circuits with $N = 16$, $c \approx 0.35$, and $d = 0.25$.

Table S3. An increase in penetrance facilitates access to a new genotype network. The fraction of shortest mutational sequences that reach a new genotype network is significantly higher when single mutations increase penetrance as much as possible than when they only preserve an alternative phenotype (Wilcoxon signed-rank test). For each combination of values for N, c and d, and for each of 5×10^3 initial random genotypes, we created 10^3 mutation sequences of each kind.

N	c	d	Fraction of mutation sequences that reach the new genotype network. Mean ± SE for mutations that:		p-value
			Increase penetrance maximally	Preserve alternative phenotype	
8	0.4	0.25	0.201 ± 0.004	0.106 ± 0.002	$< 2.2 \times 10^{-16}$
		0.125	0.164 ± 0.003	0.087 ± 0.002	$< 2.2 \times 10^{-16}$
	0.3	0.25	0.175 ± 0.003	0.145 ± 0.002	$< 2.2 \times 10^{-16}$
16	0.35	0.25	0.394 ± 0.005	0.19 ± 0.002	$< 2.2 \times 10^{-16}$
	0.25	0.125	0.296 ± 0.004	0.191 ± 0.003	$< 2.2 \times 10^{-16}$
		0.25	0.325 ± 0.004	0.213 ± 0.003	$< 2.2 \times 10^{-16}$
20	0.3	0.25	0.463 ± 0.005	0.225 ± 0.003	$< 2.2 \times 10^{-16}$
	0.2	0.1	0.329 ± 0.004	0.21 ± 0.003	$< 2.2 \times 10^{-16}$
		0.25	0.377 ± 0.004	0.253 ± 0.003	$< 2.2 \times 10^{-16}$
		0.5	0.422 ± 0.004	0.29 ± 0.003	$< 2.2 \times 10^{-16}$
	0.1	0.25	0.271 ± 0.003	0.253 ± 0.003	6.3×10^{-13}
